# Duality of Valproic Acid Effects on Inflammation, Oxidative Stress and Autophagy in Human Eosinophilic Cells

**DOI:** 10.3390/ijms241713446

**Published:** 2023-08-30

**Authors:** Goksu Uzel, Ece Oylumlu, Lubeyne Durmus, Ceren Ciraci

**Affiliations:** Molecular Biology and Genetics Department, Istanbul Technical University, 34469 Istanbul, Turkey; uzelgoksu@gmail.com (G.U.); eceoylumlu2@gmail.com (E.O.); lubeyneyuruk@gmail.com (L.D.)

**Keywords:** valproic acid, human eosinophils, eosinophilic leukemia, autophagy, NLRP3, oxidative stress

## Abstract

Eosinophils function in rapid innate immune responses and allergic reactions. Recent research has raised the possibility that the histone deacetylase inhibitor valproic acid (VPA) may be a promising therapeutic agent for treatment of allergic responses and certain cancers. However, its effects on eosinophils remain unclear. Utilizing the EoL-1 human eosinophil cell line as a model, we investigated the effects of VPA on oxidative stress- and autophagy-mediated immune responses. We found that VPA induced reactive oxidative species (ROS) generation and eosinophil activation without affecting cell viability. Moreover, VPA treatment suppressed the negative regulator of antioxidant transcription factor Nrf2, which is known to activate antioxidant defense. Interestingly, VPA was able to increase autophagic markers, as well as NLRP3 and NLRC4 mRNA activation, in Eol-1 cells in a dose-dependent manner. Collectively, our results indicate that VPA could increase the severity of allergic responses, and if so, it clearly would not be a suitable drug for the treatment of allergic reactions. However, VPA does have the potential to induce autophagy and to regulate the inflammatory responses via inflammasome-driven caspase-1 deactivation in a dose-dependent manner.

## 1. Introduction

Eosinophils are granulocytes containing granular structures in the cytoplasm that are found in the subgroup of leukocytes. After maturation in the bone marrow, they either enter the circulation in the peripheral blood or settle in the organs. Eosinophils represent 1–4% of white blood cells in the peripheral blood and are found in the primary and secondary lymphoid organs, as well as lamina propria of the gastrointestinal (GI) tract under homeostasis in a healthy body. Under pathological conditions, their numbers in the tissues and the blood rapidly increase. Although they are involved in innate immunity, they bridge the gap between innate and adaptive immunity. Eosinophils have a pivotal role in parasitic infections, asthma, gastrointestinal disorders, and allergic reactions, especially in the respiratory tract [1,2].

During the development of innate immune responses, pattern recognition receptors (PRRs) are important for the first encounter. Downstream signaling pathways are activated through ligand–receptor interaction, resulting in the activation of transcription factors, the expression and assembly of inflammasome complexes, and the maturation and secretion of various cytokines and chemokines [3]. Eosinophils express and store a large number of cationic proteins, cytokines, growth factors, chemokines, and mediators in their granules to generate a rapid immune response to allergens or parasites [1]. The release of cytokines and chemokines affects the site of inflammation by governing the survival, activation, and migration of various cells, thereby promoting immune responses [4]. Because the mechanisms of eosinophil responses to stress are not well known, the potential effects of different therapeutic agents on eosinophils need to be examined. Research over past decades has focused on the elucidation of new therapeutic agents, as well as targets for the modulation of innate immune responses. Therefore, in this study, we focused on the responses of eosinophils to alternative therapeutic modalities.

Valproic acid (VPA) is a branched short-chain fatty acid obtained from valeric acid, an essential compound of the plant *Valeriana officinalis* [5]. VPA is used as an anticonvulsant drug for the effective treatment of different types of neurological disorders, such as epileptic seizure and bipolar disorder [6]. VPA has also been reported as a histone deacetylase inhibitor (HDACi) that modulates gene expressions by inhibiting histone deacetylation [7]. As a HDACi, VPA has promising potential in cancer therapy, as it can mediate the expression of important genes involved in anti-tumor immunity. Numerous studies have shown that VPA suppressed the proliferation of prostate cancer cells through the induction of autophagic cell death [8]. Another study reported that, upon VPA treatment, autophagic cell death was observed as a result of oxidative stress in glioma cells [9]. In a different study, VPA treatment was demonstrated to suppress the activation of mouse mast cells in allergic reactions [10].

Under normal circumstances, reactive oxygen species (ROS) production is balanced by the antioxidant system; however, when the balance is disturbed by diseases/pathogens or drugs, increased intracellular ROS production gives rise to oxidative stress [11]. Oxidative stress is of great importance for numerous conditions, such as aging; neurodegenerative, cardiovascular, and inflammatory diseases; and cancer [12]. Hence, cells attempt to reduce this toxicity by developing a series of antioxidant responses to maintain homeostasis. On the one hand, the main regulator of antioxidant responses is the antioxidant transcription factor nuclear factor erythroid 2-associated factor 2 (Nrf2) for the expression of cytoprotective proteins [13,14]. On the other hand, autophagy is another mechanism responsible for maintaining homeostasis in cells against a variety of stress sources [15]. Cells remove damaged, misfolded, unnecessary, or nonfunctional cell components through lysosomal degradation in response to cellular stresses, such as nutrient deficiency or high levels of ROS. A variety of proteins and ever-evolving pathways are involved in the initiation of autophagy, the formation of the autophagosome, and the degradation of components within the autophagosome for the maintenance of autophagy [16,17,18]. Taken together, these studies suggest the stress-related effects of VPA on several different pathologies and cell types; however, our knowledge of the impacts of VPA on eosinophils is lacking. Therefore, in this study, we investigated the inflammation, autophagy, and oxidative stress in VPA-induced human eosinophilic cell line and we report for the first time VPA‘s binary effects on human eosinophil-like cells, which may lay the groundwork and provide new insights for future in vivo animal models of allergy, asthma, and airway hyperresponsiveness, as well as ex vivo human studies.

## 2. Results

### 2.1. VPA Induced Cellular ROS Generation without Altering Cell Viability in Eol-1 Cells

**Figure 1:**
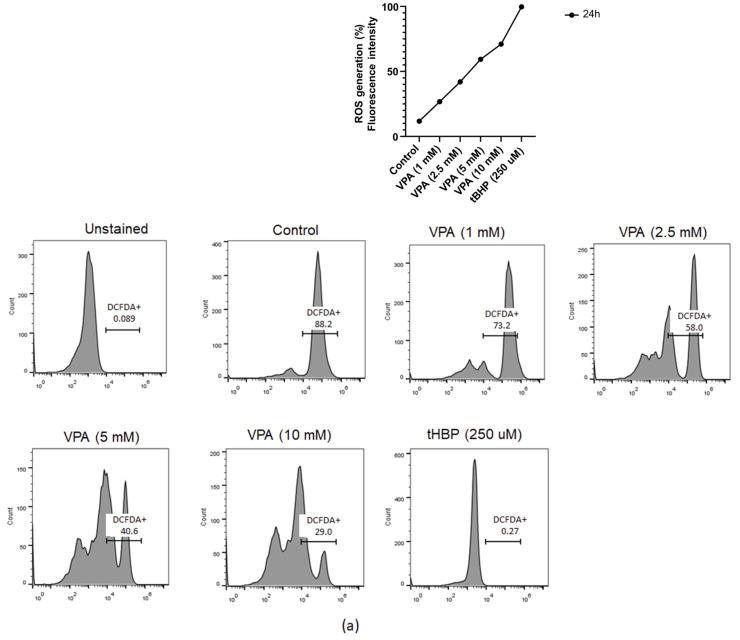

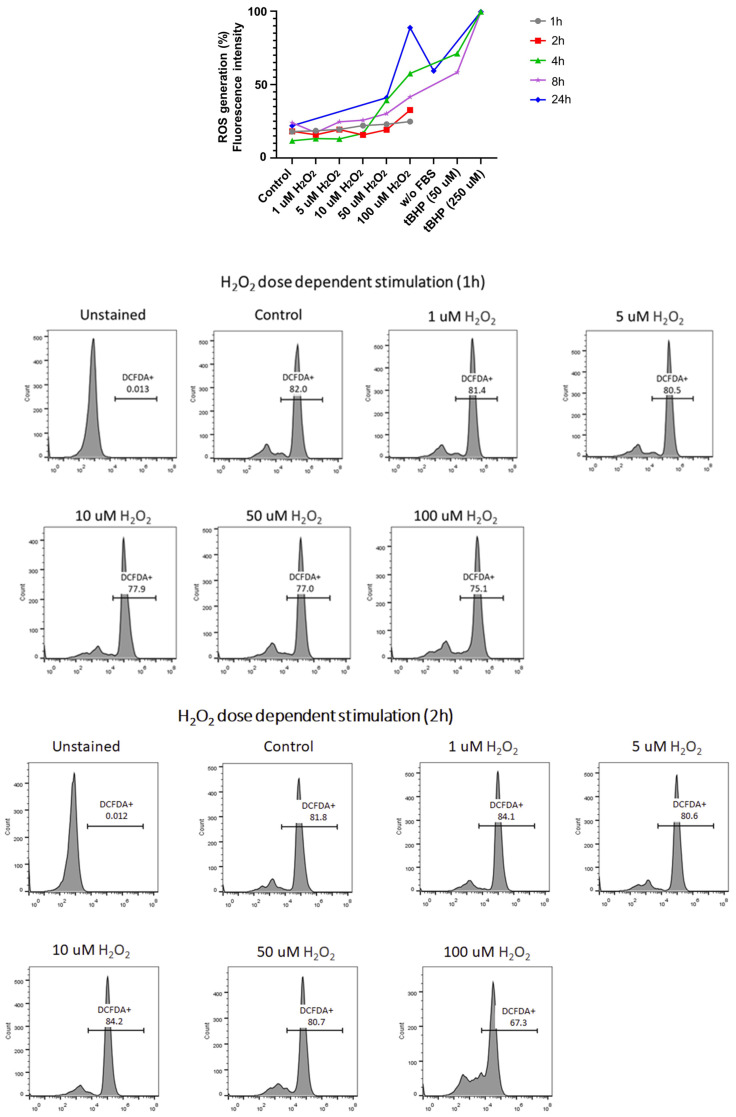

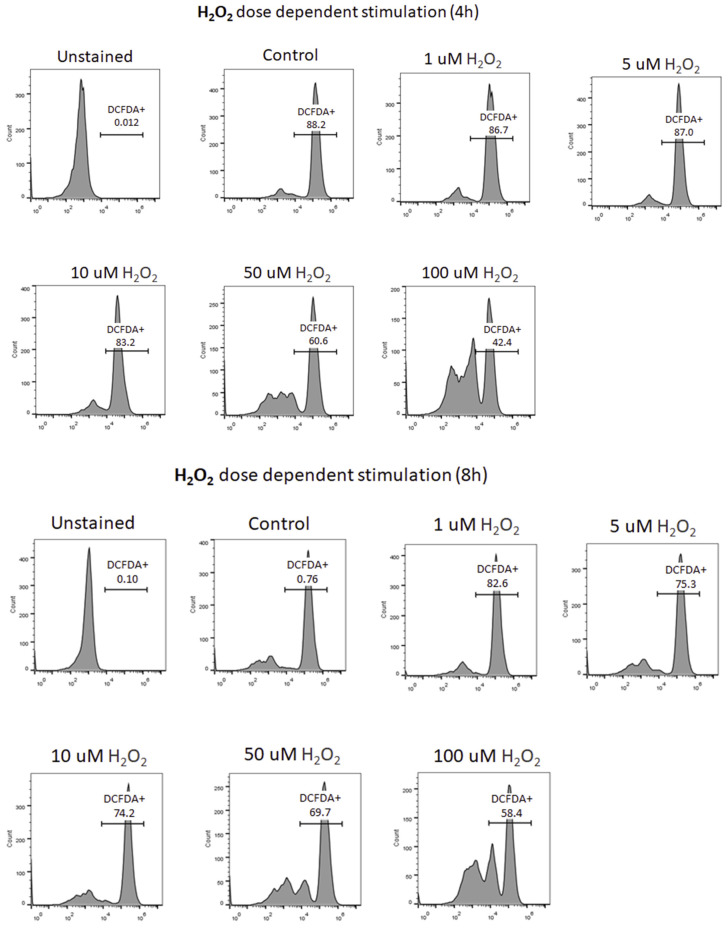

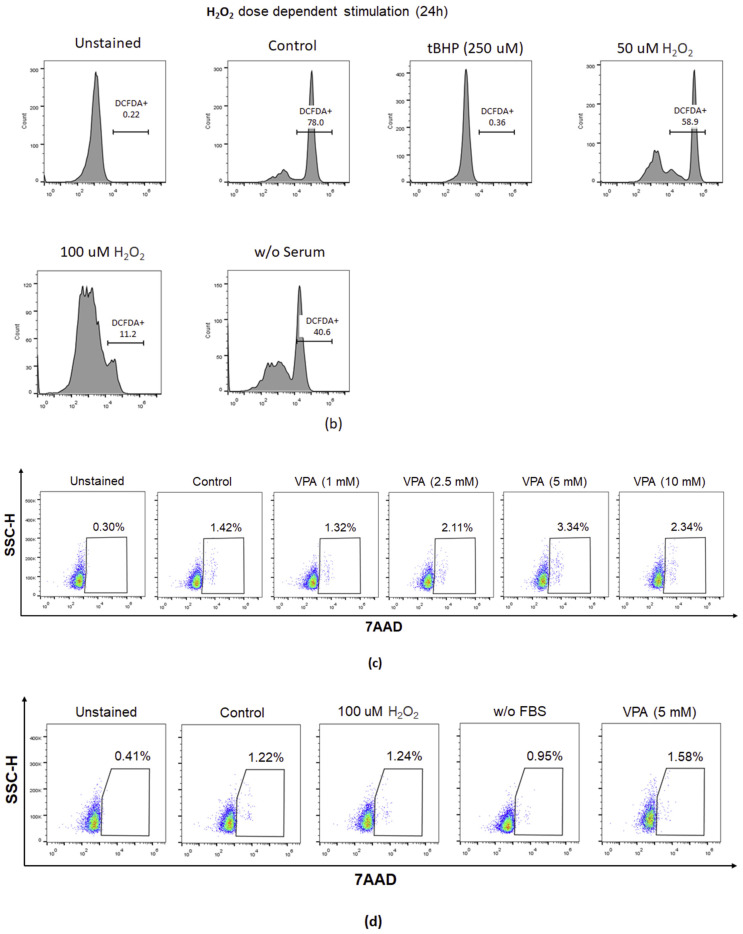
VPA treatment increased the ROS generation in a dose-dependent manner without affecting the Eol-1 cells’ viability. (**a**) DCFDA staining was performed to measure the cellular ROS in live cells by flow cytometry in Eol-1 cells treated with 1 mM, 2.5 mM, 5 mM, and 10 mM VPA for 24 h and histograms; or (**b**) with 5-µm, 10-µm, 20-µm, 50-µm, and 100-µm H_2_O_2_ for 1 h, 2 h, 4 h, 8 h, and 24 h and cultured in FBS-free (w/o FBS) media for 24 h and histograms. (**c**) Representative flow cytometry pseudocolor plots of EoL cells that were treated with VPA at various concentrations (1, 2.5, 5, and 10 mM) for 24 h; (**d**) Eol-1 cells were treated for 24 h with oxidative stress-inducing conditions (treatment with 100 µm H_2_O_2_), treated with 5 mM VPA, or cultured in FBS-free (w/o FBS) media and stained with 7AAD to analyze the viability. Experiments were carried out in triplicate. The results are representative of two independent experiments. SSC-H: side scatter height, AAD: aminoactinomycin D.

The human eosinophilic leukemia cell line (EoL-1) has been utilized as a model to investigate the influence of VPA on eosinophilic functions [19] due to its comparability to human primary eosinophils [20], which are among the least abundant cell populations in blood. Even though, VPA has been examined in the context of neurobiology, cellular stress, and cancer in multiple studies [6,8,9]; its regulatory roles, particularly at a cellular level, in eosinophils, which are involved in allergy and asthma, are largely unknown. Thus, to better understand VPA’s ability to induce ROS generation in Eol-1 cells, we tested the dose effects of VPA on ROS production by 2′,7′-dichlorofluorescein diacetate (DCFDA) staining. For this purpose, Eol-1 cells were treated with 1 mM, 2.5 mM, 5 mM, and 10 mM VPA for 24 h. Intracellular ROS levels increased with VPA treatment in a dose-dependent manner at 24 h post-treatment (Figure 1a). Two additional treatment groups that are known to induce oxidative stress or cellular stress in cells were also included in the experiments: hydrogen peroxide (H_2_O_2_)-stimulated cells and fetal bovine serum (FBS) free media (w/o FBS)-grown cells. While treatment with H_2_O_2_ has been shown to trigger several pathways depending on its concentration and cell type [21], culturing cells in FBS-free media for 24 h has been reported to increase cellular stress [22] and also autophagy [22,23] due to an insufficient growth environment. Therefore, on one side, the time and dose effects of H_2_O_2_ stimulation were determined using 1 µM, 5 µM, 10 µM, 50 µM, and 100 µM doses and 1-h, 2-h, 4-h, 8-h, and 24-h time points to test the induction of oxidative stress without exerting any cell death. On the other side, cells were cultured in FBS-free media for 24 h to address whether EoL-1 cells underwent autophagy in the absence of serum. Expectedly, treatment with 100 µM H_2_O_2_ increased ROS generation by 65.8% and serum-free media by 37.4%, while 5 mM VPA increased ROS generation by 47.6%, which were same when compared to the non-treated Eol-1 cells at 24 h (Figure 1a,b). These results suggested that VPA treatment induced ROS generation in a similar manner to already established mechanisms. Therefore, these three oxidative stress-inducing conditions were used for further analysis throughout this study. Next, we investigated Eol-1 cell viability, as excessive ROS production gives rise to cell death [21]. Interestingly, oxidative stress-inducing conditions (Figure 1c) and increasing doses of VPA treatment did not significantly affect the viability of Eol-1 cells (Figure 1d). These results indicated that VPA induced ROS generation in Eol-1 cells without altering cell viability; therefore, it might have potential advantages over more potent chemicals with severe side effects as a therapeutic drug.

### 2.2. VPA Induced the Activation of Eol-1 Cells

**Figure 2:**
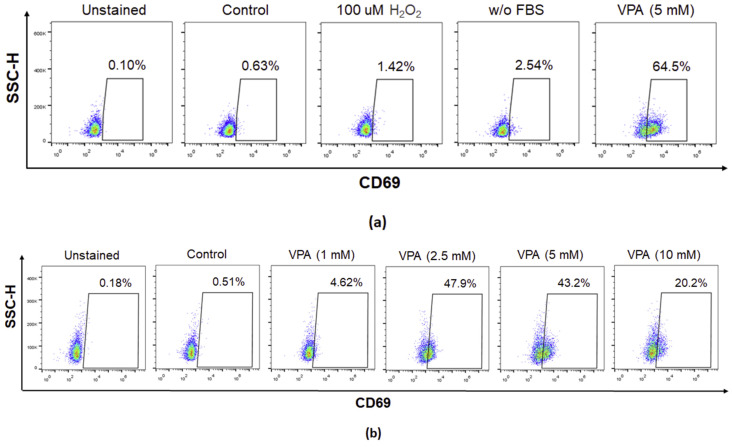
VPA treatment increased the CD69 expression in Eol-1 cells. (**a**) Representative flow cytometry pseudocolor plots of Eol-1 cells treated with 100 µm H_2_O_2_ and 5 mM VPA and cultured in FBS-free (w/o FBS) media for 24 h; or (**b**) treated with VPA at various concentrations (1, 2.5, 5, and 10 mM) and stained with CD69 to validate cell activation. Experiments were carried out in triplicates. The results are representative of two independent experiments. SSC-H: side scatter height.

Next, we determined whether VPA treatment activated EoL-1 cells following ROS generation. To this end, the surface expression of the eosinophil activation marker CD69 was analyzed by flow cytometer after VPA treatment. Although the effects of 100 µM H_2_O_2_ and serum free media exposure were negligible on the expression of CD69, 5 mM VPA significantly increased the CD69 expression in Eol-1 cells compared to non-treated cells (Figure 2a). Indeed, CD69 expression reached the highest level in Eol-1 cells upon 2.5 mM VPA treatment (Figure 2b). These results suggested that VPA activated Eol-1 cells independently of ROS generation.

### 2.3. VPA Induced the Activation of Nrf2-Dependent Antioxidant Protection

**Figure 3:**
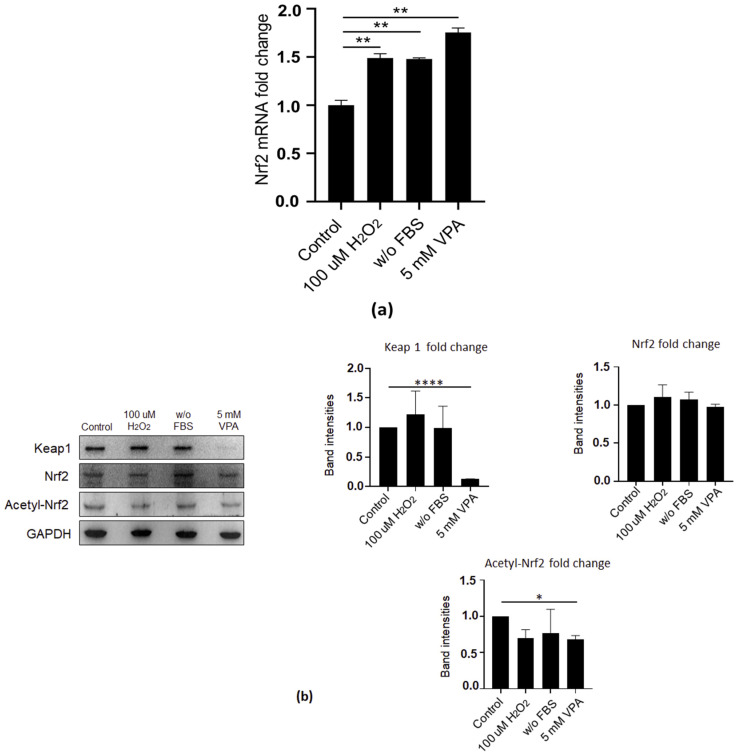

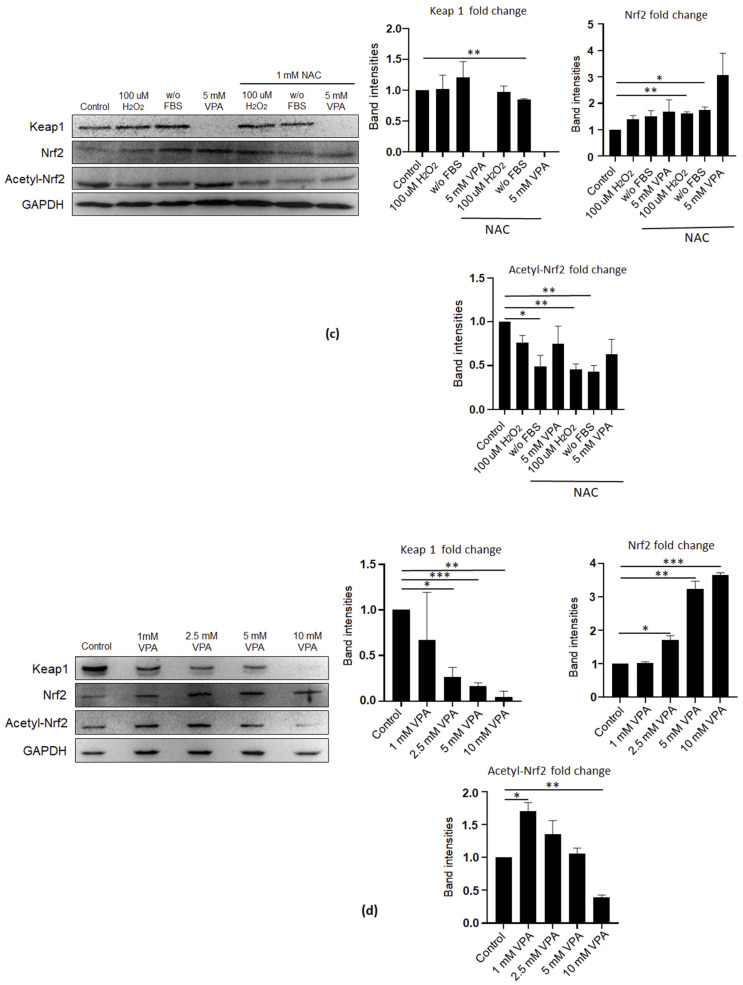
VPA treatment suppressed Keap1, the negative regulator of Nrf2, potentially leading to the activation of antioxidant responses. (**a**) Nrf2 mRNA expression in Eol-1 cells treated with 100 µm H_2_O_2_ and 5 mM VPA and cultured in FBS-free (w/o FBS) media for 24 h. Gene expression was normalized to HPRT as an internal control to calculate the fold changes. Values represent the mean ± SD of two independent experiments. Student’s *t*-test shows the significant difference between stimulated and non-stimulated cells. * *p ≤* 0.05, ** *p ≤* 0.01, *** *p* ≤ 0.001, **** *p* ≤ 0.0001. (**b**) Immunoblot analysis measured the protein levels of Keap1, Nrf2, and acetyl-Nrf2 in Eol-1 cells treated with 100 µm H_2_O_2_ and 5 mM VPA and cultured in FBS-free (w/o FBS) media for 24 h or (**c**) after 24 h of incubation, 1 h treatment with 1 mM NAC; or (**d**) treated with VPA at various concentrations (1, 2.5, 5, and 10 mM) and band intensities. GAPDH was used as an internal control. Experiments were carried out in triplicate. The results are representative of three independent experiments.

Under normal conditions, ROS production is kept in balance in cells. The antioxidant transcription factor Nrf2 is localized in the cytoplasm with Keap1 protein, which mediates Nrf2 degradation. When the balance is disrupted, increased ROS production leads to oxidative stress. Oxidative stress then gives rise to the dissociation of Nrf2–Keap1 complex, and Nrf2 is translocated to the nucleus. Then, acetylation of Nrf2 increases the transcription possibilities of the target antioxidant proteins to eventually reduce the toxicity and maintain homeostasis [11,24]. Therefore, we evaluated the antioxidant system proteins to better understand VPA’s effects on EoL-1 cells. First, we tested the oxidative stress and serum-free conditions, as well as VPA stimulation, all of which significantly upregulated the mRNA level of Nrf2 in Eol-1 cells (Figure 3a). Second, we determined the protein levels of Nrf2, acetylated Nrf2 (acetyl Nrf2), and Keap1 and showed that Keap1 protein levels were markedly decreased by only VPA treatment in Eol-1 cells. Interestingly, Nrf2 protein levels did not significantly change, while the protein levels of acetylated Nrf2 were consistently low under all tested conditions compared to Nrf2 (Figure 3b). To further verify the effect of VPA-mediated ROS production, we treated the cells with N-acetyl-L-cysteine (NAC), which is a commonly used antioxidant known to inhibit ROS production [25]. VPA suppressed Keap1 protein expression regardless of NAC treatment. While NAC restored the effects of H_2_O_2_ and serum-free media on Nrf2 protein, it did not affect Nrf2 protein levels when cells were treated with VPA. In contrast to its restorative effects on Nrf2, NAC treatment decreased the levels of acetylated Nrf2 under oxidative stress-inducing conditions (Figure 3c). Notably, we did not determine any effects of NAC per se on non-treated cells, and all tested proteins showed a similar expression profile to that of the control cells.

We then investigated the importance of increasing doses of VPA on antioxidant protection and clearly showed that it significantly decreased Keap1, whereas it increased the Nrf2 protein levels in a dose-dependent manner in Eol-1 cells. Moreover, the acetylated Nrf2 levels were upregulated with VPA at lower doses, reaching the highest level at ≥1 mM VPA treatment (Figure 3d). Collectively, these results suggested that VPA activated the antioxidant system through the suppression of Keap1 and increased the transcription possibilities of antioxidant proteins by antioxidant transcription factor Nrf2.

### 2.4. Autophagy Is Activated by VPA Treatment in Eol-1 Cells

ROS accumulation in cells is associated with myriad pathologies [26] because they can react with cellular components and destroy cell compounds, such as proteins, nucleic acids, lipids, and organelles. In such conditions, the autophagic pathway is activated to eliminate damage and maintain homeostasis in response to stress [27]. Therefore, we examined whether VPA can induce autophagy in EoL-1 cells by focusing on the pathways that have important roles in autophagy signaling. First, the MAPK/Akt/mTOR pathways were examined as the upstream effectors of autophagy. Although activation of the MAPK pathway indicates autophagosome formation and could be used as a marker for autophagy [28], it may not be sufficient per se to confirm autophagy, as the activation of MAPK pathways is also implicated in several pathophysiological events in the cell that could lead to cell survival, proliferation, cell death, etc.

**Figure 4:**
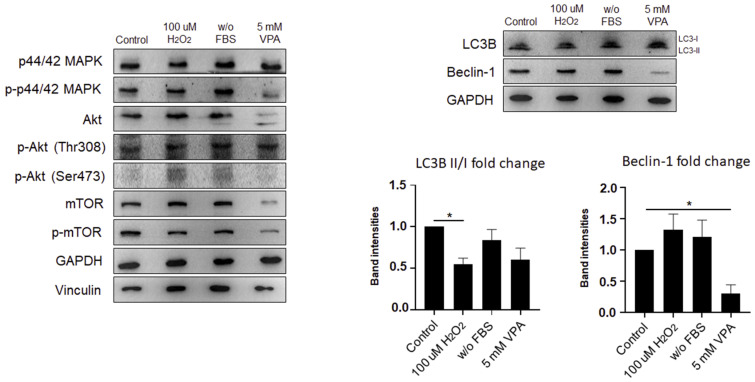

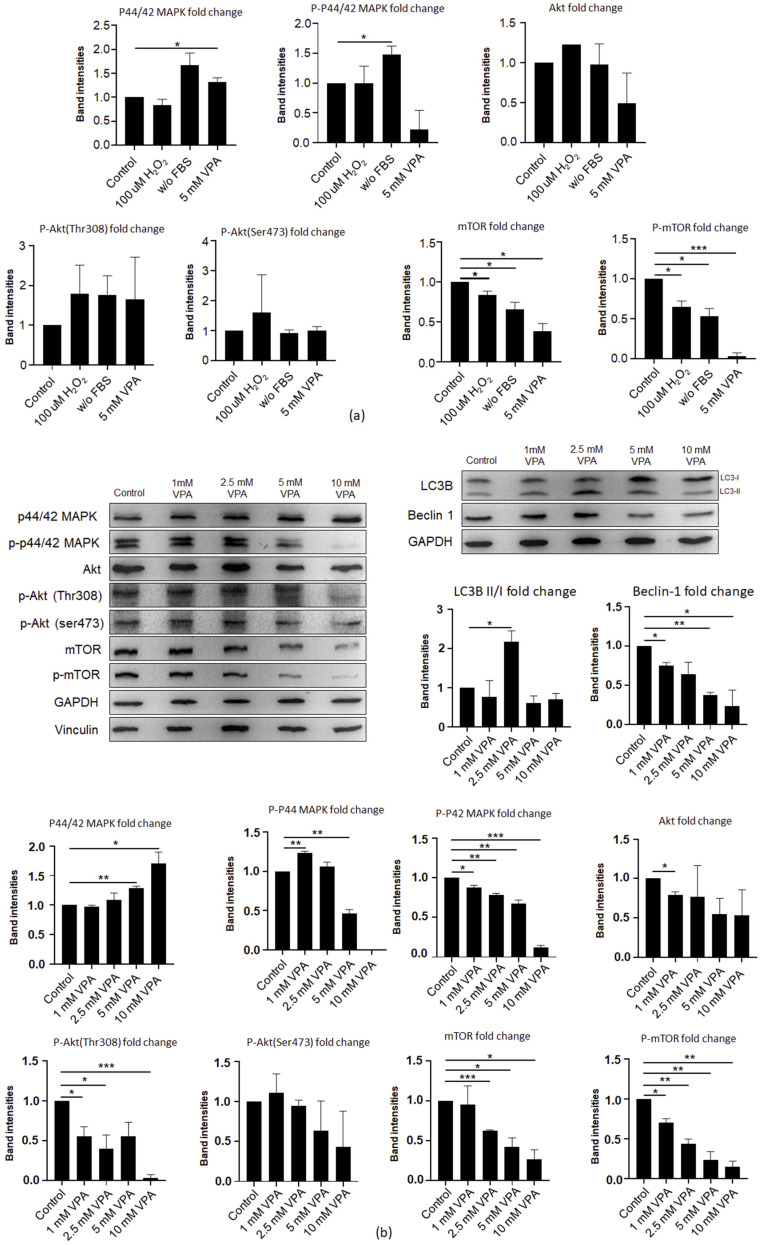
VPA treatment activated the autophagy through the MAPK pathway. (**a**) The protein levels of upstream pathways of autophagy (p44/42 MAPK, Akt, mTOR) and markers of autophagy (LC3B, beclin 1) were determined by immunoblotting in Eol-1 cells treated with 100 µm H_2_O_2_ and 5 mM VPA and cultured in FBS-free (w/o FBS) media for 24 h; or (**b**) treated with VPA at various concentrations (1, 2.5, 5, and 10 mM) and band intensities. GAPDH was used as an internal control. Experiments were carried out in triplicate. The results are representative of two independent experiments. Values represent the mean ± SD of two independent experiments. Student’s *t*-test shows the significant difference between stimulated and non-stimulated cells. * *p ≤* 0.05, ** *p ≤* 0.01, *** *p* ≤ 0.001.

Here, we observed that, while protein expression of phosphorylated p44/42 MAPK (p-p44/42 MAPK) was reduced by 5 mM VPA, culturing in serum-free media increased the levels of p-p44/42 MAPK protein in Eol-1 cells (Figure 4a). Interestingly, the dose of VPA had significant effects on the regulation of p44/42 MAPK protein, which was upregulated with increasing doses; however, p-p44/42 MAPK was markedly diminished at higher concentrations of VPA (Figure 4b). These results suggested that VPA inhibited autophagy by deactivating MAPK signaling when cells were treated with higher doses of VPA; however, lower doses of VPA (up to 2.5 mM) were sufficient to activate the autophagy marker MAPK pathway.

In the next step, we sought to investigate the changes in the Akt signaling pathway, which mediates the phosphorylation of mTOR, resulting in the inhibition of autophagy. Thus, activation of Akt and mTOR signaling pathways negatively regulates autophagy and can be used as a negative marker of the autophagy pathway [29,30,31]. While Akt phosphorylation on Ser473 (p-Akt (Ser473)) increased in both H_2_O_2_-treated and serum-free media grown cells, 5 mM of VPA treatment did not exert any impact on phosphorylated Akt (Figure 4a). Next, we detected that Akt expression only slightly decreased with 1 mM VPA treatment. Similar to MAPK signaling, VPA stimulation decreased the protein levels of p-Akt for both Ser473 and Thr308 residues at higher doses (Figure 4b).

Since its discovery, the mTOR molecule has drawn considerable attention for its involvement in growth, metabolism, and disease [32]. Most importantly, mTOR has been reported to play roles in cow’s milk allergy-associated behavioral and immunological deficits [33]. Last, the PI3K-AKT-mTOR signaling pathway has been shown to be at the crossroad of allergic asthma and cataracts [34]. Therefore, we reasoned that VPA-mediated activation of autophagy in eosinophils might involve mTOR protein as well. We showed that, while mTOR and phosphorylated mTOR (p-mTOR) levels were comparable in H_2_O_2_-treated and serum-free media grown cells (Figure 4a), 5 mM VPA treatment significantly decreased both mTOR and p-mTOR protein expression. When we further tested the effects of increasing doses of VPA on mTOR, we detected a slight diminishment for both phosphorylated and unphosphorylated protein levels with 2.5 mM VPA (Figure 4b). Concomitant with MAPK and Akt signaling, these results suggested that 2.5 mM VPA activated the upstream pathway of autophagy in Eol-1 cells in a manner that did not involve mTOR.

To further examine VPA’s ability to induce autophagy in eosinophilic leukemia cells, we determined the commonly used markers of the autophagy pathway: LC3B II/I and Beclin 1. The cytosolic form LC3-I transforms into LC3-II and colocalizes in the autophagosome membrane. The LC3-II expression level or LC3II/LC3I ratio represents the number of autophagosomes, indicating the autophagy activation [35]. Beclin 1 is also important for the formation of autophagosome structures and serves as an upstream mediator of the autophagy pathway [36]. We found that Beclin 1 was reduced after treatment with VPA at all doses but not with the other two treatment conditions (H_2_O_2_ treatment and serum free media) that we tested (Figure 4a). Moreover, the highest levels of protein expression for LC3-II/LC3-I ratio were detected in 2.5 mM VPA-treated Eol-1 cells (Figure 4b). Taken together, our data indicated that VPA might be capable of inducing autophagy in human eosinophil model cells, and the 2.5 mM VPA dose was optimal for this induction. 

### 2.5. VPA Treatment Induced the Inflammasome Complex Formation in Eol-1 Cells

**Figure 5:**
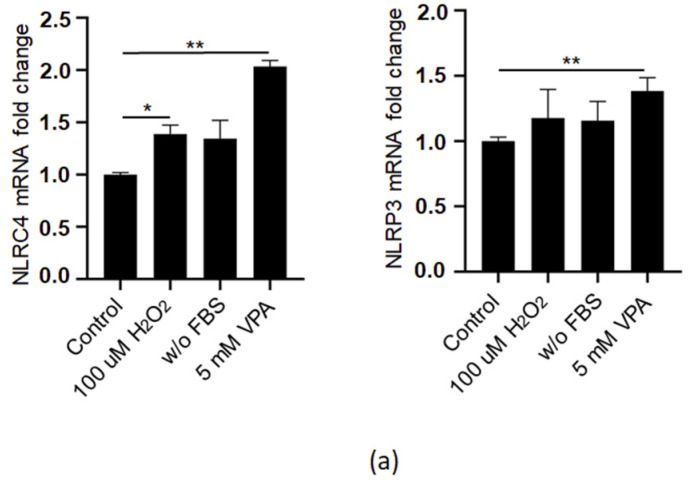

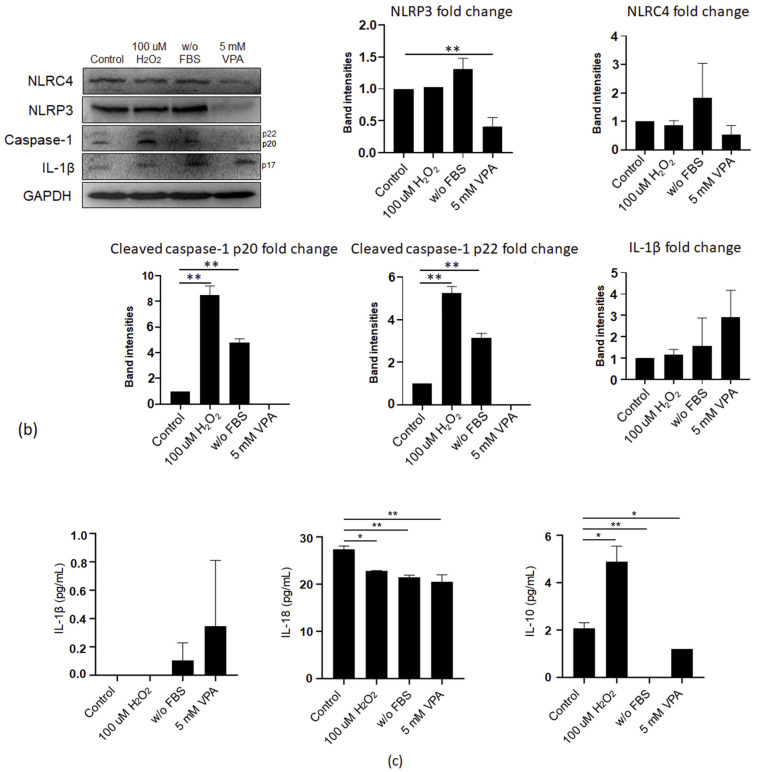

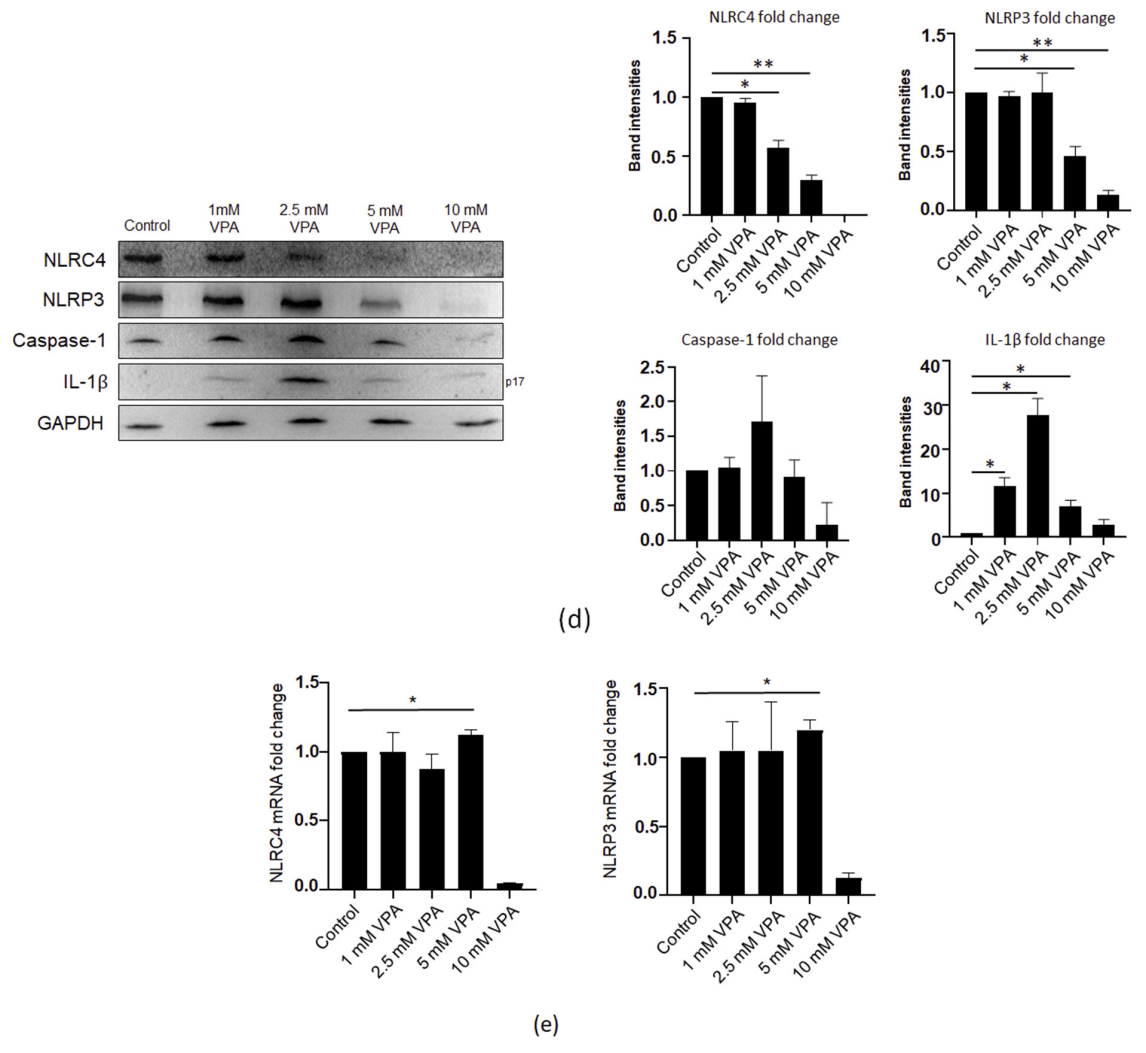
NLRC4 and NLRP3 were upregulated by lower doses of VPA (≤2.5 mM) treatment but suppressed at higher doses of VPA (≥5 mM). (**a**) mRNA expression of NLRC4 and NLRP3 in Eol-1 cells treated with 100 µM H_2_O_2_ and 5 mM VPA and cultured in FBS-free (w/o FBS) media for 24 h. Gene expression was normalized to HPRT as an internal control to calculate fold changes. Values represent the mean ± SD of two independent experiments. (**b**) The protein levels of inflammasome component proteins (NLRC4, NLRP3, caspase-1, IL-1β) were measured by immunoblotting in Eol-1 cells that were treated with 100 µM H_2_O_2_ and 5 mM VPA and cultured in FBS-free (w/o FBS) media for 24 h, and band intensities were depicted. (**c**) Pro-inflammatory cytokines (IL-1β and IL-18) and anti-inflammatory cytokine (IL-10) secretion after Eol-1 cells were treated with 100 µM H_2_O_2_ and 5 mM VPA and cultured in FBS-free (w/o FBS) media for 24 h were measured by ELISA. (**d**) Cells were treated with VPA at various concentrations (1, 2.5, 5, and 10 mM). GAPDH was used as an internal control. Experiments were carried out in triplicate. (**e**) mRNA expression of NLRC4 and NLRP3 in Eol-1 cells treated with VPA at various concentrations (1, 2.5, 5, and 10 mM). The results are presented from two independent experiments which were carried out in triplicate. Values represent the mean ± SD of two independent experiments. Student’s *t*-test shows the significant difference between stimulated and non-stimulated cells. * *p ≤* 0.05, ** *p ≤* 0.01.

Innate immune responses have to be tightly regulated to maintain homeostasis and host survival, and autophagy is one of the mechanisms that can modulate the innate immune responses. Given that immune system components can regulate autophagy through immune cells and cytokine release to protect the cells [37], we assessed the inflammatory response after induction of EoL-1 cells with VPA. We already reported that eosinophils can induce inflammatory responses through the CARD domain-containing NOD-like receptor 4 (NLRC4) which forms multimeric protein complexes called “inflammasomes” [38]. NLR proteins are members of the pattern recognition receptor (PRR) families and are known to function as NLR proteins without the formation of inflammasomes. NLRC4 and NLRP3 are the two most elucidated inflammasome-forming NLRs, and they initiate the cleavage and secretion of IL-1β and IL-18 by auto-catalytically activated caspase-1 [39]. Therefore, we initially measured the mRNA levels of NLRC4 and NLRP3 by QPCR, and they were significantly increased after 5 mM VPA treatment in Eol-1 cells (Figure 5a). Next, we determined the protein levels of NLRC4, NLRP3, caspase-1, and intracellular IL-1β by immunoblotting. Although we did not see any change in the expression of NLRC4, NLRP3, and caspase-1 under oxidative stress and serum-free conditions, these proteins were reduced after 5 mM VPA treatment of EoL-1 cells. Strikingly, intracellular IL-1β was upregulated after VPA stimulation compared to non-stimulated cells (Figure 5b). Human eosinophils express low levels of IL1β, IL18, and IL10 compared to other myeloid origin dendritic cells and macrophages [40], yet we still measured these cytokines since the extracellular IL1β, IL18, and IL10 production capacities of eosinophils under oxidative stress and serum free conditions and after VPA treatment are not known. Of all the secreted cytokines we tested, only anti-inflammatory IL-10 was potentially upregulated under oxidative stress conditions, while inflammasome-driven IL-1β and IL-18 were not detected under any condition (Figure 5c). Surprisingly, these results suggested that IL-1β might be cleaved into the mature form by a different protein in the cytoplasm since 5 mM VPA inhibited the activation of the NLRC4 and NLRP3 proteins (Figure 5b).

To further determine whether VPA is involved in the activation of the inflammasome complexes, we treated Eol-1 cells with various concentrations of VPA. While the protein expression level of NLRC4 was downregulated by all doses of VPA, NLRP3 expression was reduced by 5 mM VPA (Figure 5d). Moreover, the protein levels of intracellular cleaved IL-1β were significantly upregulated with 2.5 mM VPA (Figure 5d). Interestingly, increasing doses of VPA upregulated NLRC4 and NLRP3 mRNA expressions at 5 mM VPA; however, they were downregulated at 10 mM VPA, suggesting that VPA might have different transcriptional regulatory functions as a histone acetylase inhibitor (Figure 5e). Collectively, the results from these experiments indicated that VPA might have induced inflammasome complex formation without affecting the secretion of inflammatory cytokines in Eol-1 cells. The dose of VPA may be of great importance, changing the direction that eosinophils take in response to various stress stimuli. The higher doses of VPA activate EoL-1 cells, suppressing autophagy and inflammatory responses, whereas lower doses of VPA may induce autophagy and immune responses via inflammasomes.

## 3. Discussion

The histone deacetylase inhibitor valproic acid is widely used for bipolar disorder and epileptic seizures, as well as to treat various diseases, such as cancer [7,41]. One recent study revealed VPA as a promising therapeutic agent against allergic responses, as it suppressed the mouse mast cell activation [10]. However, VPA has been demonstrated to induce oxidative stress and autophagy, which may narrow down its therapeutic index and prevent its use in a larger set of epigenetically driven diseases [8,42].

Eosinophils are innate immune cells that have key effector roles in responses to parasitic infections and allergic reactions [1]. They express and store a large number of cationic proteins, cytokines, growth factors, chemokines, and mediators in their granules to rapidly respond to danger signals [1]. Unfortunately, the low numbers of human eosinophils in circulating blood and the insufficient protein and mRNA yield from primary human eosinophils generate technical challenges for mechanistic studies, which require high concentrations of cellular products. Therefore, we adapted the human EoL-1 eosinophil cell line as a model due to their ability to quickly respond to wide range of stimulants and for their human eosinophilic pan-marker expression profiles [20,38]. In addition, human eosinophilic cell lines, including EoL-1, its derivative EoL-3, HL-60, AML-14, and its subclone AML14.3D10, are other available cell lines for human eosinophil studies. HL-60, AML-14, and AML14.3D10 are promyeloid leukemia cells, whereas EoL-1 cells originated from an eosinophilic lineage, therefore providing a useful in vitro model [43,44,45,46]. Due to EAD’s complexity, tissue involvement, limited numbers of animal models, as well as species-specific characteristics of eosinophil behaviors and the technical challenges of working with eosinophils are the limitations of eosinophil-involved basic research. Thus, findings from this study need to be expanded to additional studies, such as in human eosinophils from broncho-alveolar lavage, to further confirm the results.

To better understand the regulatory roles of VPA as a therapeutic agent in eosinophil-associated diseases (EADs), we evaluated VPA’s effects in comparison with oxidative stress- and serum-free conditions that are well studied stress-inducing conditions, thus serving as positive controls in the experiments conducted in this study. We also determined the importance of dose and the duration of stimulation with VPA in EoL-1 cells. First, we showed that VPA did not change the cell viability when cells were treated with increasing doses of VPA. In contrast to the studies of mast cells [10], VPA was able to upregulate the cell surface activation marker CD69, yet stress-inducing conditions did not alter the CD69 protein expression on Eol-1 cells.

Elevated ROS generation has been linked to the activation of antioxidant responses to maintain cellular homeostasis for proper performance of eosinophils [14,47,48]. Therefore, we assessed the regulators of antioxidant defenses in response to VPA stimulation and found that Keap1, a negative regulator of Nrf2 transcription factor, was suppressed, and conceivably, both the mRNA and protein expression of Nrf2 was elevated upon VPA treatment. The dose of VPA was crucial in regulating the antioxidant response in EoL-1 cells, as 2.5 mM VPA upregulated acetylated Nrf2, which has been shown to increase the transcription possibilities of Nrf2 and thus target gene expression [49].

Oxidative stress has been shown to affect the activation of the autophagy pathway to regulate cell survival and homeostasis [50]. While p44/p42 MAPK (ERK1/2) is a positive regulator of autophagy, the Akt and mTOR pathways negatively regulate the autophagy pathway [28,29,30]. Because all these studies were conducted in the context of cancer or neurobiology, we addressed whether VPA can induce autophagy in eosinophil-like cells that are involved in allergy, asthma, and EADs. Given that the PI3K-AKT-mTOR signaling pathway lies in the intersection of allergic asthma and cataract [34], we analyzed the effector molecules of these pathways, including p44/p42 MAPK, p-p44/p42 MAPK, Akt, p-Akt Ser473, p-Akt Thr308, mTOR, and p-mTOR, after VPA treatment. Although higher doses of VPA (≥5 mM) suppressed both phosphorylated and unphosphorylated effector signaling molecules, with the exception of p44/p42 MAPK, lower doses of VPA (≤2.5 mM) upregulated p-p44/p42 MAPK, p-Akt Ser473. Importantly, we showed that lower doses of VPA upregulated autophagy marker LC3B II/I ratio (conversion of LC3-I to LC3-II). 

Autophagy and innate immune responses modulate each other to protect the cell [37]. Therefore, we examined the innate immune responses through NLRC4 and NLRP3 inflammasome complex formation. Similar to autophagy signaling, VPA induced the inflammasome components NLRP3 and caspase-1, as well as intracellular IL-1β, but not NLRC4 at lower doses of VPA (≤2.5 mM) without affecting the secretion of inflammatory cytokines in Eol-1 cells. 

Here, we showed that VPA induced ROS overproduction, eosinophil activation by CD69 expression, Nrf2/Keap1-dependent antioxidant defenses by suppression of Keap1 expression, activation of the autophagy pathway, and activation of the inflammasome complex formation at ≤2.5-mM VPA doses. The activation of Eol-1 cells by VPA poses the question of whether VPA should be used as a drug in allergic responses, as it may actually increase the severity of allergic reactions. Overall, however, our findings raise the possibility of new agents for the treatment of other eosinophil-related diseases, most notably eosinophilic leukemia. Finally, further mechanistic studies using in vivo animal models, as well as ex vivo human studies, could establish a framework for elucidating the reciprocal relationship between signaling pathways that are critical for eosinophil survival and tissue homeostasis.

## 4. Materials and Methods

### 4.1. Cell Culture and Stimulations

The Eol-1 human eosinophilic cell line was used as a model cell line. Eol-1 cells were cultured in RPMI 1640 medium (PAN-Biotech GmbH, Aidenbach, Germany) supplemented with 10% heat-inactivated fetal bovine serum (FBS), 2 mM glutamine, 1 mM sodium pyruvate, 0.1 mM nonessential amino acids, 100 U/mL penicillin, 100 μg/mL streptomycin, and 10 mM HEPES and incubated at 37 °C and 5% CO_2_. Eol-1 cells (1.6 × 10^6^ cells/mL) were stimulated with valproic acid at different doses (1 mM, 2.5 mM, 5 mM, and 10 mM) for 24 h in a 24 well plate. To determine the optimum time and dose of positive control for oxidative stress, Eol-1 cells (1.6 × 10^6^ cells/mL) were stimulated with H_2_O_2_ (Sigma-Aldrich, St. Louis, MO, USA ) at different doses (5 µm, 10 µm, 20 µm, 50 µm, 100 µm) for different times (1 h, 2 h, 4 h, 8 h, and 24 h). As a second positive control for cell stress, Eol-1 cells (1.6 × 10^6^ cells/mL) were cultured in serum-free RPMI 1640 media. For the further experiments, two different conditions were used: (1) Eol-1 cells were stimulated with 100 µm H_2_O_2_ and 5 mM VPA and cultured in FBS-free media for 24 h,; and (2) Eol-1 cells were stimulated with valproic acid at different doses (1 mM, 2.5 mM, 5 mM, and 10 mM) for 24 h. After the stimulation, cells were collected and lysed. To inhibit ROS formation, Eol-1 cells were stimulated with 100 µm H_2_O_2_ and 5 mM VPA and cultured in FBS-free medium for 24 h, and then they were treated with 1mM N-acetyl cysteine (NAC) (Abcam, Cambridge, UK) for 1 h according to the manufacturer’s instructions. Cells were collected and lysed after stimulation.

### 4.2. Real-Time RT-PCR

Total RNA was isolated from samples (three wells from 24-well plates, 3 replicates per each treatment) using RNAquous© (Ambion, Austin, TX, USA) according to the manufacturer’s instructions. All RNA samples were DNase treated with DNA-Free (Ambion, Austin, TX, USA) according to the manufacturer’s instructions before quantitative PCR. The primers that were used for quantitative real-time RT-PCR were hNrf2 primers (F 5′-GAGAGCCCAGTCTTCATTGC-3′, R 5′-TGCTCAATGTCCTGTTGCAT-3′), hNLRC4 primers (F 5′-GTGTTCTCCCACAAGTTTGA-3′, R 5′-AGTAACCATTCCCCTTGGTC-3′), hNLRP3 primers (F 5′-CTTCCTTTCCAGTTTGCTGC-3′, R 5′-TCTCGCAGTCCACTTCCTTT-3′) and hHPRT 1 primers (F 5′-GACCAGTCAACAGGGGACAT-3′, R 5′-AACACTTCGTGGGGTCCTTTTC-3′) as housekeeping genes using QuantiTect SYBR Green RT-PCR (Qiagen, Waltham, MA, USA) to determine mRNA expression levels. Each RT-PCR reaction was performed as previously described [38,51,52]. The mRNA levels for the target gene were corrected to those of the housekeeping gene (HPRT) and then were calculated by subtracting their corresponding cycle threshold (Ct) before and after stimulation using the following formula:

Before stimulation,

ΔCtcontrol = Cttarget gene control − CtHPRT control (1)

After stimulation,

ΔCtstimulated = Cttarget gene stimulated − CtHPRT stimulated(2)

The fold change in mRNA was determined by: Fold change 2Ct(stimulated) − Ct(control). Experiments were performed at least twice, and one representative experiment is depicted. Results are expressed as fold-changes in expression of stimulated cells relative to non-stimulated cells.

### 4.3. Detection of Intracellular Reactive Oxygen Species

The ROS formation in Eol-1 cells was measured by the 2′,7′ –dichlorofluorescein diacetate (DCFDA) cellular ROS assay. Eol-1 cells were seeded with RPMI (without phenol red) at 1 × 10^6^ cells/mL per well in a 96-well plate. Following 1-2-4-8-24 h(s) stimulations, cells were stained with 20 µM DCFDA and incubated for 30 min at 37 °C. The DCFDA stain can diffuse into the cell and be deacetylated by esterases, then being oxidized by reactive oxygen species into DCF. DCF is fluorescent and detected with fluorescein (FITC) at 485-nm/535-nm wavelengths using flow cytometry. The tert-butyl hydrogen peroxide (tbHP) used as a positive control.

### 4.4. BCA Protein Assay

A Thermo Scientific Pierce BCA Protein Test Kit was used to detect the protein concentrations of lysed cells (Thermo Fisher Scientific, Waltham, MA, USA). Cells were lysed with RIPA buffer and were centrifuged for preclearance. Supernatants were used as protein samples. The absorbance of the standards and unknown samples was measured at 562-nm wavelength using a spectrometer. 

### 4.5. Immunoblotting

Eol-1 cell lysates (30–50 µg) were prepared in a Laemmli buffer containing SDS and denatured at 95 °C for 10 min. Proteins were separated in 7.5%–10%–12% SDS-PAGE polyacrylamide gels and on polyvinylidene difluoride (PVDF) membranes (Bio-Rad Laboratories, Hercules, CA, USA). The PVDF membrane was blocked with 5% skim milk (Sigma Aldrich, St. Louis, MO, USA) and then incubated in primary antibodies overnight at 4 °C for protein detection. The primary antibodies that were used for protein detection were anti-Nrf2 (STJ, London, UK), anti-Nrf2 (Acetyl K599) (STJ, London, UK), Anti-Keap1 (CST, Danvers, MA, USA), anti-Akt (CST, Danvers, MA, USA), anti-phospho Akt (ser473) (CST, Danvers, MA, USA), anti-phospho Akt (Thr308) (CST, Danvers, MA, USA), anti-p44/42 MAPK (CST, Danvers, MA, USA), anti-phospho p44/42 MAPK (CST, Danvers, MA, USA), anti-mTOR (CST, Danvers, MA, USA), anti-phospho mTOR (Ser2448) (CST, Danvers, MA, USA), anti-LC3B (CST, Danvers, MA, USA), Anti-beclin 1 (CST, Danvers, MA, USA), anti-NLRC4 (Biolegend, San Diego, CA, USA), anti-NLRP3(CST, Danvers, MA, USA), anti-caspase-1 (p20–p22) (Abcam, Cambridge, UK), anti-cleaved IL-1β (CST, Danvers, MA, USA), anti-GAPDH (STJ, London, UK) (used as a housekeeping protein), and anti-vinculin (CST, Danvers, MA, USA) (used as a housekeeping protein). After incubation with secondary antibodies, which were HRP-conjugated anti-rabbit (CST, Danvers, MA, USA) and anti-mouse (CST, Danvers, MA, USA), the membrane was visualized by electrochemiluminescence (ECL; Roche, Mannheim, Germany) using the ChemiDoc XRS+System (Bio-Rad Laboratories, Hercules, CA, USA). All band intensities were calculated with densitometric data, which were acquired and translated using Image Lab software (Bio-Rad Laboratories, Hercules, CA, USA). 

### 4.6. Cytokine Measurement

Supernatants from Eol-1 cells were collected to determine the levels of secreted extracellular cytokines by ELISA. Nunc MaxiSorp 96-well plates were coated with purified anti-human antibodies specific for the antigen of interest and incubated overnight at 4 °C. The wells were washed with PBS-T and blocked with 10% blocking solution (sterile PBS containing 10% FBS) for 1 h at room temperature. The blocking solution was removed, and then samples and standards (by serial dilution) were added to the wells and incubated overnight at 4 °C. The wells were washed with PBS-T. Biotin-conjugated anti-human antibodies specific to the antigen of interest were dispensed into wells and incubated for 1 h at room temperature. The plates were washed with PBS-T. HRP-linked avidin D was added to each well and incubated for 30 min at room temperature. Then, the plate was washed with PBS-T. TMB Peroxidase Substrate and TMB Peroxidase Substrate Solution B (Thermo Fisher Scientific, Waltham, MA, USA) were mixed at a 1:1 ratio and added to each well. After the color changes were observed in the wells, the reaction was stopped with 1 N HCl. The absorbance was measured at 450-nm wavelength using a spectrometer. Data were analyzed using GraphPad Prism software, version 8. Purified anti-human antibodies and biotin-conjugated anti-human antibodies of IL-1β, IL-18, IL-5, IL-13, and IL-6 (Biolegend, San Diego, CA, USA) were used for ELISA.

### 4.7. Flow Cytometry

Eol cells stained with PerCp anti-human 7AAD (BioLegend, San Diego, CA, USA) and APC anti-human CD69 (BioLegend, San Diego, CA, USA) were assessed by an Accuri C6 flow cytometer (BD Biosciences, Franklin Lakes, NJ, USA) and analyzed with FlowJo software, version 7.1 (Tree Star Inc., Ashland, OR, USA). Eol-1 cells were initially gated on the basis of size and granularity using FSC-H/SSC-H by first removing debris and doublet cells using FSC-A/FSC-H. Single cells were subgated using CD69(APC) or PerCp anti-human 7AAD. Percentages within the gates indicate the proportion of CD69(APC)- or 7AAD-expressing cells in the Eol-1 cell population. SSC-H: side scatter height, FSC-A: forward scatter area, FSC-H: forward scatter height.

### 4.8. Statistical Analyses

Statistical analyses were performed using Student’s unpaired two-tailed *t*-test or two-way ANOVA (* *p* ≤ 0.05, ** *p* ≤ 0.01, *** *p* ≤ 0.001, and **** *p* ≤ 0.0001).

## Data Availability

The data presented in this study are all contained within the article.
